# Pan-Genome Analysis and Expression Profiling of *HIPP* Gene Family in Cassava

**DOI:** 10.3390/genes17020136

**Published:** 2026-01-27

**Authors:** Zhanming Xia, Jiazheng Zhao, Changyi Wang, Shuwen Wu, Yuwei Zang, Dayong Wang, Shousong Zhu, Yi Min

**Affiliations:** 1 Department of Biotechnology, School of Life and Health Sciences, Hainan University, Haikou 570228, China; 2 Laboratory of Biopharmaceuticals and Molecular Pharmacology, School of Pharmaceutical Sciences, Hainan University, Haikou 570228, China; 3 One Health Cooperative Innovation Center, Hainan University, Haikou 570228, China; 4 Key Laboratory of Tropical Biological Resources, Hainan University, Haikou 570228, China; 5 School of Breeding and Multiplication (Sanya Institute of Breeding and Multiplication), School of Tropical Agriculture and Forestry, Hainan University, Sanya 572025, China; 6 National Key Laboratory of Tropical Crop Biobreeding, Sanya 572025, China

**Keywords:** cassava, pan-genome, *HIPP* gene family, abiotic stress response, biotic stress response

## Abstract

**Background:** Cassava (*Manihot esculenta* Crantz) ranks as the sixth largest food crop worldwide and serves as an important cash and energy crop. Heavy-metal-associated isoprenylated plant proteins (HIPPs) are metallochaperones involved in metal homeostasis and stress adaptation in vascular plants. However, research on the identification and function of HIPPs in cassava has been poorly explored. **Methods:** This study conducted a pan-genome-wide investigation to identify and characterize MeHIPPs in 31 cassava accessions. Subsequent analyses examined their physicochemical properties, subcellular localization, phylogeny, Ka/Ks, chromosomal localization, synteny, gene structure, and cis-acting elements. Additionally, the expression profiles of *MeHIPPs* in different tissues and cell subsets and under different stress conditions were analyzed using transcriptome data and quantitative real-time polymerase chain reaction (qRT-PCR). **Results:** A total of 59 *MeHIPP* pan-genes were identified, including five core genes, 22 softcore genes, 17 dispensable genes, and 15 private genes, which were unevenly distributed on chromosomes. Based on phylogenetic analysis, these genes were classified into five major subgroups. Evolutionary analyses indicated that segmental duplication predominated in family expansion and that most members may be subjected to purifying selection. Cis-element analysis highlighted the importance of MeHIPPs in plant adaptation to environmental stress. The expression profiles suggested widespread involvement of *MeHIPP* genes in response to *Xanthomonas phaseoli* pv. *manihotis* (Xpm) infection and drought stress. Different *MeHIPP* genes exhibited varying transcript levels in different tissues and cell subsets. qRT-PCR analysis revealed that the selected *MeHIPP* genes had distinct expression patterns under Cd stress. **Conclusions:** This study provides valuable insights into the functional characteristics of *MeHIPP* genes and their evolutionary relationships, laying a theoretical foundation for further functional research on stress resistance.

## 1. Introduction

Cassava (*M. esculenta*) is a significant tuberous root crop in the genus Manihot of the Euphorbiaceae family [1]. As a major tropical crop and the world’s sixth-largest food source, cassava is a critical staple crop, often referred to as the “king of starch” and “energy crop” [2]. In addition to food, it serves as an industrial feedstock for starch-based products, feed, pharmaceuticals, cosmetics, biopolymers, and biofuels [3,4]. Cassava is notable for its tolerance to diverse biotic and abiotic stresses, as well as its capacity for post-stress recovery, enabling its growth on infertile, drought-prone, and acidic soils [1,4]. Nevertheless, like many tropical crops, cassava routinely encounters multiple stresses during growth and development, which compromise plant vigor and ultimately reduce quality and yield [5,6]. In recent decades, rapid industrialization and unsustainable human activities have exacerbated metal contamination in soil and water [7]. Elevated concentrations of metal pollutants, especially heavy metals such as cadmium, arsenic, lead, and copper, suppress crop growth and diminish yield and quality, causing severe phytotoxicity manifested as reduced biomass, impaired photosynthesis, chlorosis, altered nutrient assimilation, disrupted water balance, and even plant death [8,9,10,11].

To mitigate heavy metal toxicity, plants have evolved a suite of complementary strategies, including immobilization of metals in the cell wall, chelation into metal-ligand complexes, vacuolar sequestration, regulation of membrane permeability and transport, and maintenance of cellular redox homeostasis [12,13]. These processes are mediated by multiple protein families or chelators that mediate metal ion chelation, transport, and subcellular partitioning to achieve detoxification and isolation. Representative examples include mitogen-activated protein kinases (MAPKs), ATP-binding cassette (ABC) transporters, cation diffusion facilitators (CDFs), phytochelatins (PCs), and metallochaperones [14,15,16,17,18].

Metallochaperones are a class of proteins with specific functions involved in plant growth, development, and stress responses, playing significant roles in metal homeostasis and heavy metal detoxification [19]. A defining structural feature is the highly conserved heavy-metal-associated (HMA) domain. Based on structural differences, metallochaperones are broadly classified into two subfamilies: heavy-metal-associated plant proteins (HPPs) and heavy-metal-associated isoprenylated plant proteins (HIPPs) [20]. HIPPs are specific to vascular plants. They contain one or two HMA domains and a conserved C-terminal isoprenylation motif, CaaX (where “C” denotes cysteine, “a” an aliphatic amino acid, and “X” any amino acid) [21]. The HMA domain binds metal ions to maintain intracellular metal homeostasis, and the isoprenylation motif serves as a key post-translational modification that mediates protein–membrane and protein–protein interactions in plants [20,22].

The physiological functions of HIPPs have been investigated across multiple species, including *Arabidopsis thaliana*, maize (*Zea mays*), wheat (*Triticum aestivum*), rice (*Oryza sativa*), and tomato (*Solanum lycopersicum*), revealing broad roles in plant responses to biotic and abiotic stresses, particularly heavy metal stress, and underscoring their importance for growth, development, and environmental adaptation [23,24,25,26,27]. In *Arabidopsis*, AtHIPP26 interacts with ATHB29 to mediate drought responses, and the expression of *AtHIPP20*, *AtHIPP22*, *AtHIPP26*, and *AtHIPP27* in the cadmium-sensitive yeast mutant *Δycf1* enhances tolerance to Cd [20,28]. AtHIPP33 functions as a selective autophagy cargo receptor by binding cadmium and interacting with ATG8e through an AIM (ATG8-interacting motif), thereby promoting autophagy-mediated vacuolar sequestration and enhancing cadmium detoxification [29]. In addition, AtHIPPs modulate cytokinin oxidase/dehydrogenase (CKX) degradation and respond to cytokinin negative-feedback signals to maintain cellular homeostasis [23]. In rice, knockout of *OsHIPP17* increases Cd accumulation in roots, and *OsHIPP41* is strongly induced by cold and drought stress [21,26]. Heterologous expression of *OsHIPP24* in the wild-type yeast (*Saccharomyces cerevisiae*) strain BY4741 elevates copper and cadmium accumulation, while both overexpression and knockout of *OsHIPP24* impair rice growth [30]. In wheat, transient silencing of *TaHIPP1* enhances resistance to stripe rust, suggesting that *TaHIPP1* acts as a negative regulator of disease response [25]. In grapevine, VvHIPP21 interacts with VvHOS1 and negatively regulates cold and drought responses [31]. In quinoa, CqHIPP34 interacts with CqNAC79 to enhance drought tolerance, promote chlorophyll accumulation, and strengthen antioxidant capacity [32].

Research on HIPPs has primarily focused on *Arabidopsis* and several major crops. To date, 45, 59, 66, and 34 *HIPP* genes have been identified in *Arabidopsis*, rice, maize, and tomato, respectively [21,27,33]. However, *HIPP* genes in cassava have not yet been fully characterized. Traditional gene family identification typically relies on a single reference genome, whereas pan-genomic analyses can reveal family members present within a species’ gene pool but absent from the reference. In cassava, comprehensive genome-wide identification and functional characterization of the *HIPP* gene family are still lacking. To address this gap, we performed a genome-wide identification of cassava *HIPP* genes using the pan-genome of 31 cassava accessions. We further analyzed their molecular characteristics, evolutionary relationships, chromosomal distribution, gene duplication events, promoter cis-elements and expression patterns. This work lays a foundation for understanding the *HIPP* gene family in cassava and provides candidate genes for future genetic improvement and stress-resilience breeding.

## 2. Materials and Methods

### 2.1. Identification and Physicochemical Properties of HIPP Genes in Cassava

The genome sequences, protein sequences, and genome annotation files (general feature format version [GFF], gene transfer format) for 31 different cassava accessions were downloaded from the cassava comprehensive multi-omics database CassavaDB (https://nature.hainanu.edu.cn/CassavaDB/) (accessed on 15 September 2025). *Arabidopsis* HIPP sequences were obtained from The Arabidopsis Information Resource (TAIR, http://www.arabidopsis.org/) (accessed on 18 September 2025). The hidden Markov model (HMM) files for the heavy-metal-associated (HMA) domain (PF00403) were downloaded from the Pfam database (http://pfam.xfam.org/) (accessed on 27 August 2025) [34]. Using the HMMER 3.0 software suite subroutine HMMsearch, HIPP candidate proteins were identified by searching full-length protein sequences against the HMA domain model. Additionally, homologous genes in cassava were obtained through BLAST searches using the amino acid sequences of the *Arabidopsis* HIPP family as a reference in TBtools v2.376 [35]. The results from both methods were merged, and duplicate entries were removed. Candidate sequences were subsequently submitted to the InterPro database [36], retaining only those containing the HMA domain. To further refine the selection, MeHIPP candidate proteins were screened using the C-terminal isoprenylation motif CaaX as a criterion, utilizing the PrePS tool [37].

The physicochemical properties and subcellular localization of MeHIPPs were predicted using ExPasy [38] and DeepLoc 2.1 [39].

### 2.2. Phylogenetic Analysis of the HIPP Gene Family

HIPP family members in the yam (*Dioscorea rotundata*) and rubber tree (*Hevea brasiliensis*) genomes were also identified using HMMER and BLASTp (https://www.ncbi.nlm.nih.gov/assembly/) (accessed on 21 September 2025). A total of 59 HIPP sequences from rice were obtained from the Rice Genome Annotation Project (RGAP, https://rice.uga.edu/) (accessed on 18 September 2025), while HIPPs from poplar (*Populus trichocarpa)* were obtained from previously published studies [21]. Based on the HMMsearch output, amino acid sequences corresponding to the HMA domains were extracted from HIPPs of cassava, *Arabidopsis*, rice, poplar, yam, and rubber tree. Multiple sequence alignments of all HMA domain sequences were performed using the MUSCLE algorithm integrated in MEGA 7.0 [40]. Phylogenetic analysis was subsequently conducted using the maximum likelihood (ML) method in IQ-TREE v3.0.1 [41], with the best-fit substitution model (Dayhoff) automatically selected. The robustness of the phylogenetic relationships was evaluated using 1000 bootstrap replicates. The resulting phylogenetic tree was visualized and annotated using iTOL [42].

### 2.3. Homology and Presence/Absence Variation Analysis of the HIPP Gene Family in Cassava

Orthologous gene groups (OGGs) of *HIPP* genes among 31 cassava accessions were identified using OrthoFinder v2.5.5 under the following settings: MSA infers gene trees, DIAMOND aligns sequences, and FastTree builds trees [43]. Following a systematic pan-genome naming convention, the *HIPP* OGGs across the 31 cassava genomes were uniformly renamed as *MeHIPP*. To determine syntenic relationships of *HIPP* genes within and between species, the cassava reference genome AM560 was aligned with the genomes of 11 other plant species using the Diamond tool. Synteny relationships were analyzed using the One Step MCScanX—SuperFast module in TBtools (using an e-value threshold of 1 × 10^−10^. and visualized using the Dual Synteny Plot and Advanced Circos tools [35,44]. The presence/absence variation (PAV) heatmap of the *MeHIPP* family members across the 31 cassava genomes was generated using the pheatmap package in R.

### 2.4. Ka/Ks Calculation

The coding sequences (CDS) and protein sequences of MeHIPP family members from the 31 cassava genomes were compared, and the nonsynonymous (Ka) and synonymous (Ks) substitution rates between gene pairs were calculated using KAKS_Calculator 3.0 [45]. To ensure robustness and reliability, paired data with Ks < 0.005 and Ka/Ks ratio < 0.01 were excluded. The resulting data were visualized using the R packages ggplot2 (v4.0.1), pheatmap (v1.0.13), and ggridges (v0.5.7) [46].

### 2.5. Chromosomal Localization, Cis-Element Prediction, and Conserved Motif Analysis

Chromosomal lengths and *MeHIPP* genomic coordinates were extracted from the cassava GFF files using TBtools, and the chromosomal distribution of *MeHIPP* genes was visualized accordingly [35]. For promoter analysis, 2000 bp upstream sequences of each *MeHIPP* coding sequence (CDS) were retrieved from the cassava genome. Cis-acting regulatory elements were predicted using the PlantCARE database (http://bioinformatics.psb.ugent.be/webtools/plantcare/html/) (accessed on 4 November 2025) [47]. Conserved protein motifs were identified using the Multiple Em for Motif Elicitation website (https://meme-suite.org/meme/) (accessed on 6 November 2025) under the following settings: classic mode, any number of repetitions, and number of motifs set to 10 [48].

### 2.6. RNA-Seq Data Analysis of HIPP Genes

RNA-seq datasets from 11 cassava tissues (PRJNA324539) were obtained from the European Nucleotide Archive (ENA, https://www.ebi.ac.uk/ena/) (accessed on 11 November 2025), and datasets generated under drought stress and infection with *Xanthomonas phaseoli* pv. *manihotis* (PRJNA385393, PRJNA231851) were downloaded from the National Center of Biotechnology Information (NCBI, https://www.ncbi.nlm.nih.gov/bioproject/) (accessed on 13 November 2025) [49,50]. RNA-seq data were analyzed using the Trimmomatic, Hisat2, and featureCounts packages on the Galaxy database (https://galaxyproject.org/) (accessed on 15 November 2025) [51]. The raw counts were divided by gene length (kb) to calculate the reads per kilobase (RPK). Each gene’s RPK was divided by the sum of RPK values across all genes and multiplied by 10^6^ to yield transcripts per million (TPM). TPM values were manually calculated and log2 transformed.

### 2.7. Analysis of the Single-Cell Transcriptome of Cassava Root and Leaf

Using our single-cell sequencing maps of cassava tubers (PRJNA895163) and leaves (PRJCA019992), we examined the expression patterns of the *MeHIPP* genes at the single-cell level [52,53]. Data were analyzed on the OmicSmart platform (https://www.omicsmart.com) (accessed on 28 November 2025), and Uniform Manifold Approximation and Projection (UMAP) embeddings together with gene expression bubble plots were generated.

### 2.8. Plant Materials and Treatments

Cassava (*M. esculenta* Crantz cv. SC8) plants, a typical cassava cultivar, were transplanted from the Tropical Crops Genetic Resources Institute (TCGRI, Danzhou, China). All plants were grown on Murashige and Skoog (MS) medium for 2 to 3 months. Seedlings with comparable vigor were selected for subsequent experiments. For metal stress treatment, seedlings were exposed to 40 μM Cd^2+^. Leaves were collected at 0, 2, 4, 8, 12, 24, and 48 h, immediately snap-frozen in liquid nitrogen, and stored at −80 °C until further use.

### 2.9. RNA Extraction and Quantitative Real-Time PCR (qRT-PCR) Analysis

Total RNA was extracted from plant tissues using the RNAprep Pure Plant Kit (TIANGEN, Beijing, China), and first-strand cDNA was synthesized using the All-in-One First-Strand Synthesis Master Mix (Share-bio, Shanghai, China) according to the manufacturer’s instructions. Each 10 μL qRT-PCR reaction contained 5 μL premix (Share-bio, Shanghai, China), 0.4 μL each of forward and reverse primers, 1.2 μL cDNA, and 3 μL ddH_2_O. Gene-specific primers targeting *MeHIPP* genes were designed using NCBI Primer-BLAST (https://www.ncbi.nlm.nih.gov/tools/primer-blast/, accessed on 28 November 2025). qRT-PCR was performed on a Light Cycler 96 (Roche, Switzerland). *β-tubulin* (*TUB*) and *elongation factor 1α* (*EF1α*) served as internal reference genes [54,55]. Thermal cycling conditions were: 95 °C for 30 s; followed by 40 cycles of 95 °C for 10 s, 59 °C for 10 s, and 72 °C for 30 s; and a final extension at 72 °C for 15 s. Three biological replicates were performed, each with three technical replicates. Relative expression levels were calculated using the 2^−ΔΔCt^ method, and statistical differences were assessed with Tukey’s test [56].

## 3. Results

### 3.1. Identification of HIPP Genes in Cassava

To characterize the composition and features of the *HIPP* gene family in cassava, we performed genome-wide identification at both the reference genome and pan-genome levels. Based on the heavy-metal-associated (HMA, PF00403) domain profile characteristic of HIPPs in the Pfam database and *HIPP* gene sequences in *Arabidopsis*, we conducted the hidden Markov model searches using HMMER and obtained homologous genes in cassava using BLASTP. All candidate sequences were subsequently confirmed using InterPro to ensure the presence of the intact HMA domain. In the reference genome AM560, we identified 51 *HIPP* genes. Across the pan-genome, which includes 31 cassava accessions, we detected 59 *HIPP* pan-genes, representing a total of 1209 *HIPP* genes across different cassava accessions. The number of *HIPP* genes per accession varied from 26 to 61, reflecting considerable variation in gene presence or absence and lineage-specific gene gain or loss within this family (Figure S1).

Physicochemical analysis revealed that the HIPPs ranged from 95 to 839 amino acids in length, with molecular masses ranging from 10.36 to 90.93 kDa. The theoretical isoelectric points (pI) varied between 4.97 and 9.68. The aliphatic index ranged from 32.05 to 109.14, and the instability index ranged from 18.54 to 72.50. Only two proteins exhibited positive GRAVY values, suggesting that, overall, HIPPs are hydrophilic. Subcellular localization predictions indicated that most HIPPs are localized in the cytoplasm or at the cell membrane, with a minority targeted to the peroxisomes or the nucleus (Table S1).

### 3.2. Presence/Absence Variation and Phylogenetic Analysis of HIPP Gene Family

**Figure 1:**
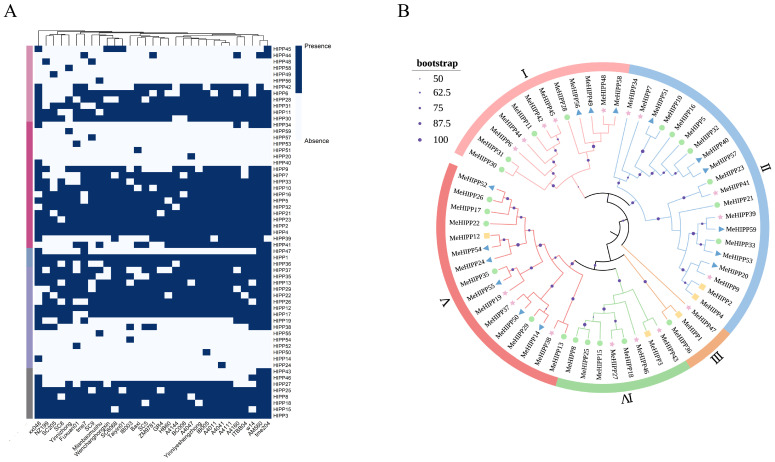
Phylogenetic and presence/absence analyses of the *MeHIPP* gene family. (**A**) Heatmap showing the presence/absence variation in *HIPP* genes across 31 cassava genomes. Dark blue indicates the presence of genes, whereas ghost white denotes the absence of genes. (**B**) Phylogenetic tree of the *HIPP* gene family in cassava. The phylogenetic tree is divided into five major clades, designated I–V. Different colors represent the various subgroups. Squares represent core genes, circles represent softcore genes, asterisks represent dispensable genes, and triangles represent private genes.

Presence/absence analysis of the identified *MeHIPP* family members indicated that the cassava *HIPP* family comprises five core genes, 22 softcore genes, 17 dispensable genes, and 15 private genes. Across the 31 cassava accessions, the relative proportions of core, softcore, and dispensable genes were comparable, whereas private genes were rare or absent in many accessions. Notably, *MeHIPP1*, *MeHIPP2*, *MeHIPP3*, *MeHIPP4*, and *MeHIPP5* were present as multiple copies in most accessions, and some genes also showed more than one copy in specific accessions. *MeHIPP1*, *MeHIPP2*, *MeHIPP3*, *MeHIPP4*, and *MeHIPP12* were present in all the cassava accessions. In contrast, certain genes exhibited accession-specific presence. For instance, *MeHIPP14* was found only in the xx048 accession, while *MeHIPP52* and *MeHIPP53* were exclusive to the Fuxuan01 accession. This suggests that these genes may be linked to cultivar-specific traits. In the AM560 reference genome, *MeHIPP14*, *MeHIPP20*, *MeHIPP24*, *MeHIPP40*, *MeHIPP45*, and *MeHIPP48*–*MeHIPP59* were not identified. Among these genes, all except *MeHIPP45* were classified as private genes, occurring only in a specific accession. *MeHIPP45* was detected in only a limited number of accessions (Figure 1A).

To elucidate the evolutionary relationships and genomic variation in the *HIPP* gene family in cassava, we performed phylogenetic analyses based on the HMA conserved domain sequences derived from the MeHIPPs. To gain broader evolutionary insights, *HIPP* family members from *Arabidopsis*, *O. sativa*, *P. trichocarpa*, *D. rotundata*, and *H. brasiliensis* were also incorporated to construct the cross-species phylogenetic tree (Figure S2). Notably, five genes, *MeHIPP28*, *MeHIPP48*, *MeHIPP49*, *MeHIPP56*, and *MeHIPP58*, exhibited significant species-specific clustering.

The maximum likelihood (ML) phylogenetic tree classified the *MeHIPP* gene family into five major clades (Figure 1B). Group II represented the largest cluster, containing 20 *MeHIPP* genes. Group V and Group I included 16 and 12 *MeHIPP* genes, respectively, while Group IV comprised eight genes, and Group III formed the smallest clade with only three *MeHIPP* members.

### 3.3. Differential Selection Pressures Acting on MeHIPP Genes in Cassava

**Figure 2:**
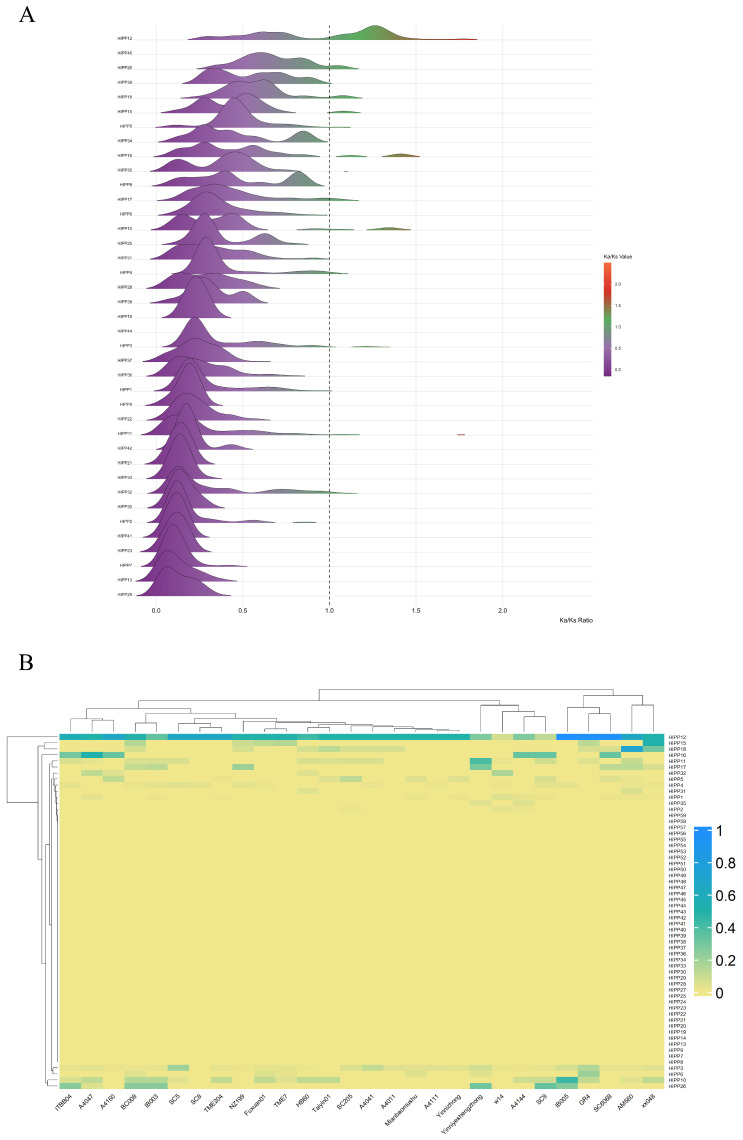
Analysis of Ka/Ks ratios of *HIPP* genes in cassava. (**A**) Ka/Ks values of *HIPP* genes across cassava genomes. (**B**) Heatmap of frequency of Ka/Ks ratios greater than 1 for each *HIPP* gene across different accessions.

The ratio of nonsynonymous (Ka) to synonymous (Ks) substitution rates (Ka/Ks) is widely used to assess the selection pressure on gene family members across species. To explore the evolutionary constraints on the *MeHIPP* gene family, we assessed the selection of *MeHIPP* family members using the ratio of nonsynonymous (Ka) to synonymous (Ks) substitution rates. Ka and Ks were calculated for each *MeHIPP* gene pair, and Ka/Ks ratios were used to infer selection. Most *MeHIPP* genes displayed Ka/Ks values between 0 and 1, indicating that the *MeHIPP* gene family in cassava is under potential signatures of strong purifying selection. This indicates that these genes may have been subject to evolutionary constraints to preserve their functional integrity and structural conservation. However, several genes displayed evidence of potential positive selection in certain cassava accessions (Figure 2A). Further heatmap analysis of genes with Ka/Ks ratios greater than 1 revealed that *MeHIPP12* exhibited consistently high Ka/Ks values across most accessions, implying that this gene has undergone potential signatures of positive selection during cassava evolution. In contrast, *MeHIPP10*, *MeHIPP11*, *MeHIPP15*, *MeHIPP16*, *MeHIPP17*, *MeHIPP18*, *MeHIPP26*, and *MeHIPP32* showed Ka/Ks > 1 more frequently in specific accessions, suggesting that these genes may be subject to potential impact of lineage-specific positive selection (Figure 2B).

### 3.4. Chromosomal Localization

**Figure 3:**
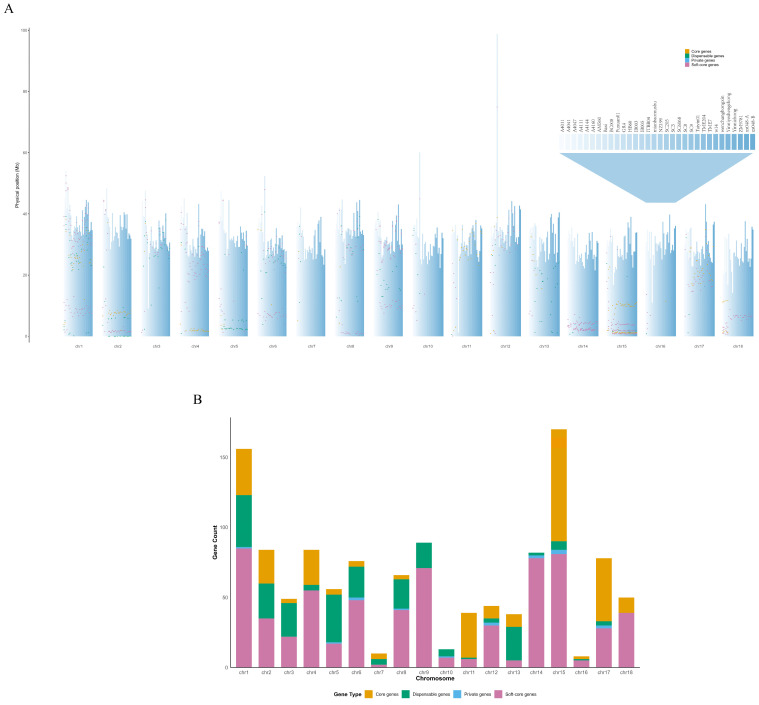
Genomic distribution of *MeHIPP* genes across 31 cassava genomes. (**A**) Genomic positions of *MeHIPP* genes on 18 chromosomes. (**B**) Per chromosome counts and proportions of the four gene categories. Orange represents core genes, pink represents softcore genes, green represents dispensable genes, and blue represents private genes.

To examine the chromosomal distribution of *MeHIPP* genes in cassava, we mapped their genomic positions across 31 cassava accessions. All *MeHIPP* genes were unevenly distributed across the 18 cassava chromosomes, with those on chromosomes 2, 14, and 15 primarily concentrated at the chromosomal ends. Chromosome 15 contained the largest number of *MeHIPP* genes (170), followed by chromosome 1 (156). Furthermore, chromosomes 9, 2, 4, 14, 17, and 6 contained similar numbers of *MeHIPP* genes, with counts of 89, 84, 84, 82, 78, and 76, respectively. In contrast, chromosomes 10, 7, and 16 harbored comparatively fewer genes, with 13, 10, and 8 genes, respectively (Figure 3A).

Softcore *MeHIPP* genes predominated across most chromosomes, particularly on chromosomes 14 (95.12%), 9 (79.78%), and 18 (78.00%). On chromosome 11, *MeHIPP* genes were mainly core genes (82.1%), revealing that these *MeHIPP* genes on this chromosome are highly conserved. In contrast, chromosomes 5 and 13 were enriched in dispensable genes (60.7% and 63.2%, respectively). Private *MeHIPP* genes accounted for a relatively small proportion of the total and exhibited an uneven chromosomal distribution (Figure 3B).

### 3.5. Synteny Analysis of MeHIPP Genes Within Cassava and Interspecific Synteny Analysis

**Figure 4:**
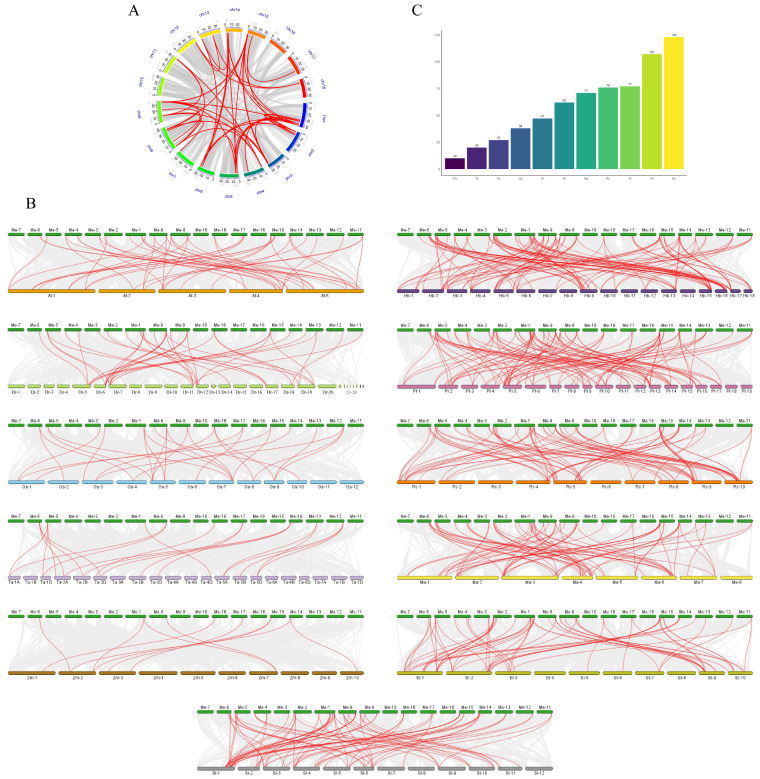
Synteny analysis of *MeHIPP* genes. (**A**) Duplicated gene pairs within *MeHIPP*. Segmentally duplicated gene pairs are connected by lines, with red lines indicating *MeHIPP* segmental duplications. Vertical lines on individual chromosomes represent tandem duplication events, and dark vertical lines denote *MeHIPP* tandem duplications. (**B**) Syntenic relationships between cassava and *Arabidopsis* (At), tomato (Sl), rice (Os), wheat (Ta), maize (Zm), yam (Dr), castor bean (Rc), poplar (Pt), rubber tree (Hb), caper spurge (El), and annual mercury (Ma). Gray lines represent all syntenic relationships, while red lines indicate those involving *HIPP* genes. (**C**) Number of syntenic *HIPP* gene pairs between cassava and other species. The abscissa represents the other species, and the ordinate represents the number of syntenic pairs.

Within a species, many genes do not exist as a single copy. Instead, they come in multiple homologous copies. These copies can perform the same or overlapping functions, even though their DNA sequences are not identical. Synteny analysis of *MeHIPP* genes in the reference genome AM560 identified at least 47 pairs of segmentally duplicated genes and two pairs of tandemly duplicated genes among the 51 *MeHIPP* members, indicating that segmental duplication was the predominant contributor to the expansion of the *MeHIPP* family (Figure 4A).

To explore evolutionary and functional relationships across species, we performed comparative synteny analyses between cassava and eleven other plants, including *Arabidopsis*, tomato, rice, wheat, maize, yam, castor bean (*Ricinus communis*), poplar, rubber tree, caper spurge (*Euphorbia lathyris*), and annual mercury (*Mercurialis annua*) (Figure 4B). The number of syntenic *MeHIPP* gene pairs varied among species, with cassava and rubber tree showing the highest number (123 pairs), followed by poplar (107 pairs). Substantial synteny pairs were also observed with castor bean (76 pairs), annual mercury (71 pairs), and caper spurge (62 pairs), reflecting the strong conservation of *HIPP* genes within Euphorbiaceae. Additionally, a considerable number of syntenic pairs (77 pairs) were identified between cassava and tomato, a dicotyledonous plant. Cassava and *Arabidopsis* shared 47 collinear pairs, fewer than those observed for the other dicotyledonous plants examined. In contrast, fewer syntenic relationships were found with monocots: 27 pairs with rice, 20 with wheat, and 10 with maize. Similarly, cassava and the tuberous monocot yam exhibited a limited number of syntenic pairs (38) (Figure 4C).

### 3.6. Conserved Motif, Gene Structure, and Cis-Acting Regulatory Elements

To further elucidate the evolutionary relationships among the *MeHIPP* genes, we analyzed their gene structures and conserved protein motifs. The number of exons per *MeHIPP* ranged from 1 to 10, with most genes containing 2 to 5 exons, suggesting that the *MeHIPP* family has undergone exon gain and loss during evolution. The majority of *MeHIPP* genes possessed 0–2 untranslated regions (UTRs) (Table S2).

To better understand the conservation and diversity of *MeHIPP* genes, ten conserved motifs (motifs 1–10) were identified in all MeHIPPs using MEME. Members belonging to the same pan-gene exhibited highly similar gene exon–intron architectures and protein motif compositions. However, there were significant differences in the number of exons among different *MeHIPP1* copies (Table S2). Representative members from different pan-genes were selected for further motif analysis, revealing comparable exon–intron structures and motif arrangements within different clades, with one or two copies of the core motif 1 retained in all MeHIPPs (Figure S3).

**Figure 5:**
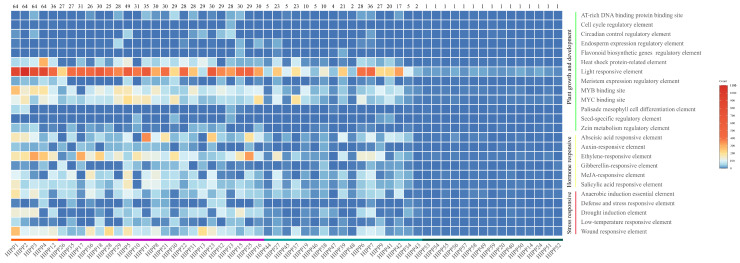
Distribution of cis-regulatory element counts across *MeHIPP* genes. The numbers inside the boxes indicate the number of cis-acting regulatory elements present in each gene. The numbers above the figure represent the total gene count per *MeHIPP* across various cassava accessions.

To explore the potential transcriptional regulatory mechanisms of the *HIPP* gene family in cassava, the promoter regions of *MeHIPP* genes were analyzed using the PlantCARE database. A total of 24 distinct cis-acting regulatory elements were identified, including 13 plant growth- and development-related cis-elements, 6 hormone-related cis-elements, and 5 stress-related cis-elements. Light-responsive elements were the most abundant and widespread, detected in all *MeHIPP* promoters, with particularly high frequencies in *MeHIPP2*. This suggests that light signaling plays a fundamental role in the regulation of *MeHIPP* transcription. The binding sites for MYB and MYC transcription factors were frequently observed. In addition, numerous hormone-related cis-elements were identified in this study. Abscisic acid (ABA) responsive elements and ethylene-responsive elements were widely distributed, while responsive elements of methyl jasmonate (MeJA) and salicylic acid (SA) were also prevalent. Additionally, several stress-related cis-elements associated with anaerobic induction, drought, low temperatures, and wounding were identified. Collectively, these findings reveal that *MeHIPP* promoters contain diverse cis-elements potentially involved in growth, hormonal, and stress-related regulatory pathways, which may respond rapidly to endogenous and exogenous stimuli and contribute to the regulation of plant development (Figure 5, Table S3).

### 3.7. Expression Patterns of MeHIPP Genes in Different Tissues and Under Different Stress Conditions

**Figure 6:**
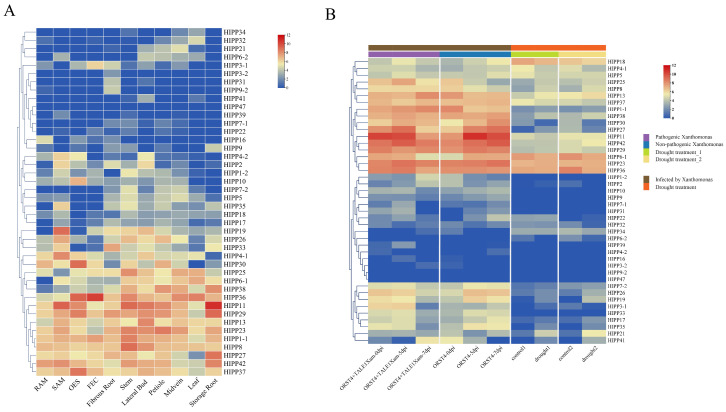
Expression profile of *MeHIPP* genes. (**A**) Eleven different tissues. (**B**) Drought stress and *Xanthomonas phaseoli* pv. *manihotis* (Xpm) infection. Drought treatment_1 and drought treatment_2 were applied to leaf material from the cassava varieties xx048 and KU50.

We analyzed bulk RNA-seq data obtained from the ENA database to profile *MeHIPP* gene expression in different tissues. The expression of different *MeHIPP* genes exhibited distinct patterns across distinct tissues. *MeHIPP1-1*, *MeHIPP8*, *MeHIPP13*, *MeHIPP23*, *MeHIPP29*, *MeHIPP37*, and *MeHIPP42* exhibited broad and relatively high expression across 11 plant tissues. *MeHIPP11* and *MeHIPP36* were not only broadly expressed but also showed notable enrichment in storage roots and friable embryogenic callus (FEC), respectively. Similarly, *MeHIPP6-1*, *MeHIPP25*, *MeHIPP27*, and *MeHIPP38* were highly expressed in the majority of tissues. Several genes displayed pronounced tissue specificity. Expression of *MeHIPP21* was strongly biased toward midvein. *MeHIPP10* was highly specifically expressed in organized embryogenic structures (OES). *MeHIPP19* was highly specifically expressed in the shoot apical meristem (SAM). In contrast, *MeHIPP7-1*, *MeHIPP22*, *MeHIPP39*, and *MeHIPP47* were generally expressed at low levels. These genes, with their varied expression profiles, may play crucial roles in plant development (Figure 6A).

To investigate the impact of various stress conditions on gene expression, we examined *MeHIPP* transcript levels under drought and pathogen stress treatments. Most *MeHIPP* genes showed expression changes in response to *X. phaseoli* pv. *manihotis* (Xpm) infection and drought stress conditions. During Xpm strain infection, *MeHIPP1-1*, *MeHIPP13*, *MeHIPP23*, *MeHIPP29*, *MeHIPP30*, *MeHIPP36*, *MeHIPP37*, *MeHIPP38*, and *MeHIPP42* maintained relatively high expression across time points, with minimal differences between pathogenic and non-pathogenic strains. In contrast, *MeHIPP6-1*, *MeHIPP11*, and *MeHIPP27* were downregulated under pathogenic infection but showed an upward trend under non-pathogenic infection. Furthermore, *MeHIPP3-1*, *MeHIPP7-2*, *MeHIPP17*, *MeHIPP19*, *MeHIPP26*, *MeHIPP33*, and *MeHIPP35* were downregulated at 7 days post-infection with the pathogenic strain but were upregulated in response to non-pathogenic strains. Notably, *MeHIPP41* showed an initial decrease, followed by an increase under pathogenic infection, but its expression gradually decreased under non-pathogenic treatment. Under drought conditions, *MeHIPP6-1*, *MeHIPP18*, *MeHIPP23*, and *MeHIPP36* maintained high and stable expression. *MeHIPP8*, *MeHIPP19*, *MeHIPP21*, *MeHIPP26*, *MeHIPP35*, *MeHIPP38*, and *MeHIPP41* were induced, with *MeHIPP21* showing the greatest induction. In contrast, *MeHIPP4-1*, *MeHIPP6-2*, and *MeHIPP30* were markedly suppressed. These results suggest that *MeHIPP* genes exhibit complex and diverse transcriptional responses under various stress conditions (Figure 6B).

### 3.8. Expression Patterns of MeHIPP Genes in Single-Cell Transcriptomes

To investigate the expression patterns of *MeHIPP* genes at the single-cell level, we analyzed single-cell RNA-seq data from cassava tuberous roots and leaves. *MeHIPP* expression varied markedly among different organs and cell types. In leaves, most *MeHIPP* genes were predominantly expressed in epidermis, companion cells, and vascular bundles. In storage roots, the spatial expression was highly heterogeneous. High expression was primarily observed in internal phloem-associated parenchyma (IPAP), vascular bundles, and endodermis clusters of the tuberous roots (Figure S4).

**Figure 7:**
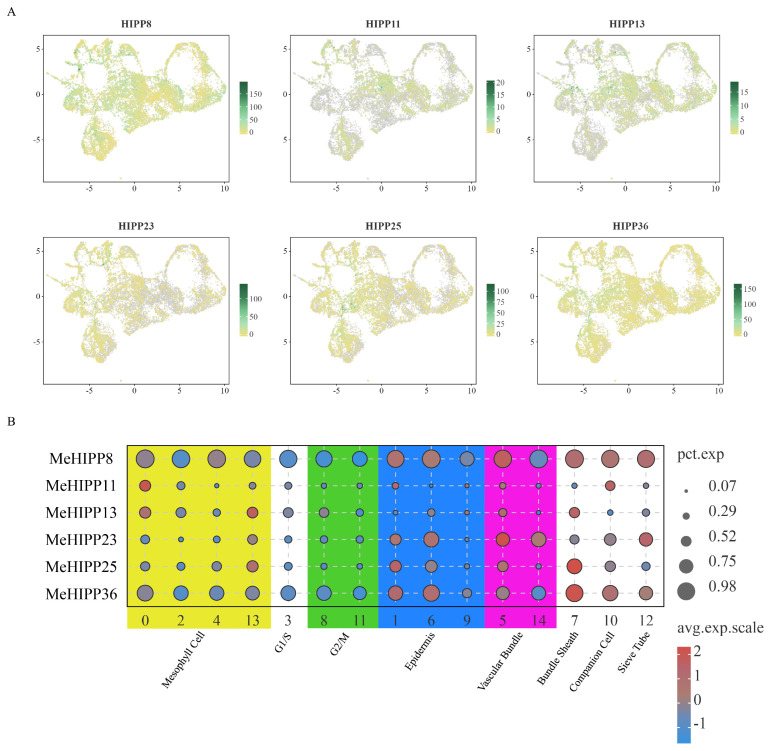
Single-cell transcriptome analysis of selected *MeHIPPs* in cassava leaves. (**A**) Uniform Manifold Approximation and Projection (UMAP) of the six *MeHIPPs.* (**B**) Bubble diagram of the six *MeHIPPs*.

Based on the systematic evolutionary analyses, we selected representative genes from each subgroup that showed consistently broad and relatively high expression levels across diverse tissues. These genes were selected for detailed single-cell analysis. In leaves, *MeHIPP8*, *MeHIPP25*, and *MeHIPP36* were mainly enriched in epidermis and bundle sheath and were expressed across a large proportion of cells. In addition, *MeHIPP8* and *MeHIPP36* displayed broad expression across other cell clusters. *MeHIPP23* was highly expressed in epidermis, sieve tubes and vascular bundles, with expression of *MeHIPP23* detected in a larger number of cells. In contrast, *MeHIPP11* and *MeHIPP13* were primarily expressed in epidermis and mesophyll cells, with high expression in companion cells and bundle sheath, respectively. However, they were detected in only a small proportion of cells compared with other selected genes (Figure 7, Table S4).

**Figure 8:**
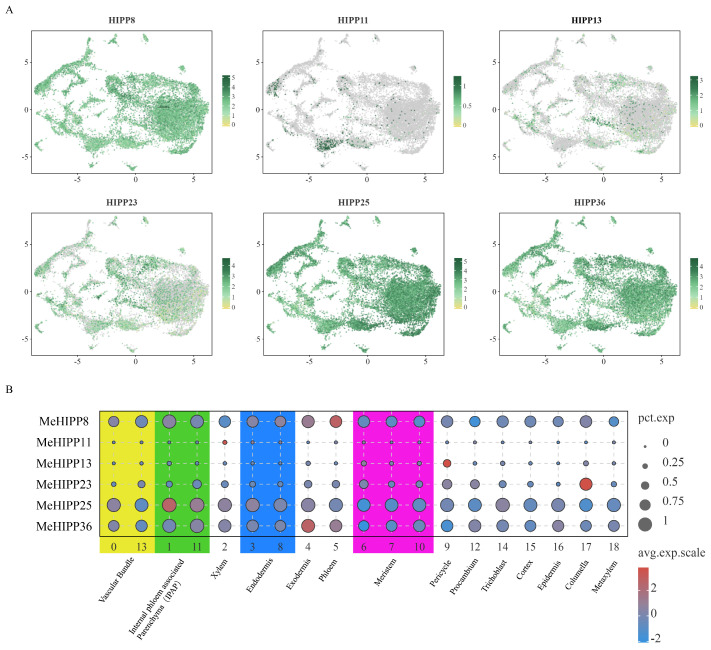
Single-cell transcriptome analysis of selected *MeHIPPs* in cassava tuberous root. (**A**) Uniform Manifold Approximation and Projection (UMAP) of the six *MeHIPPs*. (**B**) Bubble diagram of the six *MeHIPPs*.

In tuberous roots, *MeHIPP8*, *MeHIPP25*, and *MeHIPP36* were broadly expressed in multiple cell clusters. *MeHIPP8* showed high expression in phloem, *MeHIPP25* in IPAP, and *MeHIPP36* in exodermis. Additionally, *MeHIPP23* was specifically and highly expressed in columella, while *MeHIPP11* and *MeHIPP13* were specifically and highly expressed in xylem and pericycle, respectively (Figure 8, Table S4).

### 3.9. Expression Profiles of Selected MeHIPP Genes Under Cd Stress

**Figure 9:**
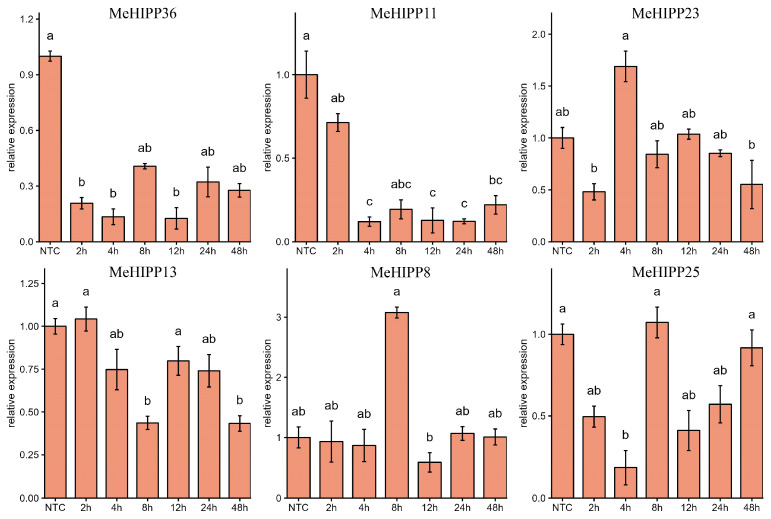
Expression of the selected *MeHIPPs* in response to cadmium (Cd) stress. The values represent the mean ± SE from three independent experiments. The horizontal axis represents the time after treatment, while the vertical axis represents the relative expression levels. Means not sharing a letter are significantly different (*p* < 0.05), for example, “a” and “b,” “ab” and “bc”.

To assess the responses of the six *MeHIPP* genes to heavy metal stress, we quantified their transcript levels using qRT-PCR. Under cadmium (Cd) stress, *MeHIPP36* was significantly downregulated. *MeHIPP11* decreased sharply at 4 h and then remained stable. *MeHIPP8* was strongly upregulated at 8 h but returned to baseline levels shortly thereafter. A biphasic pattern was observed for *MeHIPP23*, with significant downregulation at 2 h and subsequent upregulation at 4 h. *MeHIPP13* exhibited a progressive decline over time. Notably, *MeHIPP25* displayed two distinct episodes of significant downregulation and two significant rebounds (Figure 9).

## 4. Discussion

Conventional gene family analyses are confined to a single reference genome, limiting their ability to capture the full spectrum of genetic diversity within a species. The recent pan-genomic investigation of the *bHLH* gene family in barley demonstrated the value of expanding beyond single-genome approaches and set a precedent for similar analyses in other plant species [57]. With the release of comprehensive pan-genome resources for cassava, it is now both timely and essential to conduct a pan-genomic exploration of the *HIPP* gene family, which can provide a more complete understanding of its evolutionary dynamics and functional diversity and lay the foundation for subsequent research [58]. In this study, we performed the first pan-genome-wide identification and characterization of *HIPP* genes across 31 diverse cassava accessions, revealing their genetic variation and providing preliminary insights into their potential biological functions.

The *HIPP* gene family exhibits distinct numerical diversity within the cassava pan-genome. Through domain-based HMM searches combined with BLAST-based filtering, we identified 59 *HIPP* pan-genes corresponding to 1209 distinct gene copies across the 31 accessions. Variable numbers of *MeHIPP* genes were detected across cassava accessions, ranging from 26 to 61 per accession, with the majority harboring 31 to 45 functional members per accession. Such variation may reflect genuine biological differences among accessions, while also being influenced by the accuracy and completeness of distinct genome assemblies and annotations. Notably, the genome data of the “xx048” accession is a complete telomere-to-telomere (T2T) reference genome, within which a comprehensive and complete set of *HIPP* gene members has been identified [59]. Collectively, this suggests that *HIPP* gene expansions and losses have occurred frequently within the cassava genome. The observed variation in gene number likely reflects divergent evolutionary trajectories among cassava lineages, with certain lineages undergoing multiple rounds of gene duplication, thereby contributing to the overall expansion of the *HIPP* family [58].

All the *MeHIPP* genes identified across the 31 cassava accessions were systematically grouped into 59 OGGs, which were further classified into four categories: five core genes (present in all accessions), 22 softcore genes (frequency ≥ 90%), 17 dispensable genes (present in at least two accessions but at ≤90% frequency), and 15 private genes (accession-specific), thereby establishing a comprehensive evolutionary classification for the 59 *HIPP* OGGs. MeHIPPs were classified into five subgroups, with cluster II containing the largest number of members and cluster III containing the fewest. Notably, the phylogenetic analysis across multiple species revealed that *MeHIPP28*, *MeHIPP48*, *MeHIPP49*, *MeHIPP56*, and *MeHIPP58* formed distinct species-specific clusters, with *MeHIPP49*, *MeHIPP56*, and *MeHIPP58* identified as private genes. This suggests that this lineage may have undergone cassava-specific expansion or acquired new genes. Within the *MeHIPP* gene family, 47 segmentally duplicated and 2 tandemly duplicated gene pairs were identified, exhibiting duplication patterns similar to those reported in maize and tea plant [33,60]. Such gene duplication events may enhance adaptability to environmental stress and promote evolutionary flexibility. Cross-species synteny analysis revealed that cassava shares a higher number of syntenic *HIPP* gene pairs with rubber tree, poplar, castor bean, annual mercury, caper spurge, tomato, and *Arabidopsis*, all of which are dicot species. Notably, more orthologous gene pairs were identified between cassava and other members of the Euphorbiaceae family. Although cassava shares fewer syntenic pairs with *Arabidopsis* than with other dicot species, this may be limited by the smaller genome of *Arabidopsis*. In contrast, relatively few syntenic pairs were detected between cassava and monocots, such as maize, wheat, rice, and yams. These findings suggest that *HIPP* genes may have evolved distinct and important functions in the environmental adaptation of dicots. The monocot maize *HIPP* syntenic pairs reported in a previous study appear to be specific to monocots and are absent in dicots [33]. Consistent with this observation, as a dicot species, lotus shows substantially higher conservation of collinear *HIPP* gene pairs with other dicots than with monocots [61]. Similarly, cassava exhibited markedly stronger *HIPP* synteny with dicots than with monocots, indicating that *MeHIPPs* share closer evolutionary relationships within dicots. Together, these patterns are consistent with the notion that this lineage diversified after the monocot–dicot split during HIPP family evolution, with *MeHIPPs* evolving predominantly within the dicot lineage and potentially acquiring functions relevant to environmental adaptation. Ka/Ks analyses showed that most *MeHIPP* genes exhibited ratios below 1, consistent with predominant purifying selection and suggesting the potential presence of strong functional constraints over evolutionary timescales. In contrast, a smaller subset displayed Ka/Ks ratios greater than 1, indicating possible positive selection and potential functional diversification during recent cassava evolution.

Promoter cis-regulatory element analysis of *MeHIPP* genes revealed numerous stress-responsive and hormone-related motifs, indicating that these genes may play a role in stress tolerance and growth regulation. Light-responsive elements were detected in all *MeHIPP* promoter regions, indicating a fundamental role of light signaling in the regulation of this gene family. Additionally, cis-acting elements putatively recognized by the transcription factors MYB and MYC were identified. MYB factors are known to regulate gene expression in response to heavy metal stress [62]. Accordingly, further investigation of the transcriptional regulation of *MeHIPP* genes is warranted, with MYB- and MYC-binding sites serving as primary targets for defining the core promoter motifs of these genes.

Expression profiling across organs provides a basis for elucidating the roles of *MeHIPP* genes in metal homeostasis in cassava. To date, expression patterns of the *HIPP* gene family in different tissues have been reported in wheat, tea plant, and tobacco [60,63,64]. The expression profiles of *MeHIPP* genes across different tissues revealed both constitutive and tissue-specific expression patterns, suggesting that these genes may perform diverse functions in plant development and organ-specific responses. Single-cell RNA sequencing enables the analysis of gene expression patterns at the single-cell level, providing a powerful approach for exploring plant cell growth, development, and stress responses in greater depth [65]. *MeHIPP* genes are widely expressed at relatively high levels in the leaf vascular bundles and epidermis, suggesting they may play potential roles in nutrient transport and storage, defense, and plant growth [66,67]. In storage roots, *MeHIPP* gene expression across distinct cell subsets displayed pronounced cell-type specificity, suggesting that the *MeHIPP* family may undergo expression specialization during root development and cellular differentiation. The internal phloem is embedded within the xylem of vascular bundles, and its parenchyma cells are generally considered components of the phloem system [68]. High expression of *MeHIPP* genes was observed in the internal phloem-associated parenchyma (IPAP) and vascular bundles of storage roots. *HIPP* genes have been reported to modulate the expression of genes associated with metal transport and may contribute to metal uptake and translocation [69]. We hypothesize that *MeHIPP* genes could participate in metal handling by coordinating metal ions via their HMA domains, thereby supporting metal homeostasis and transport-related functions. Moreover, *MeHIPP* genes showed broader expression across diverse leaf cell subsets than in storage roots. The preferential expression of *MeHIPP* genes within specific cell subsets implies that these genes may have shared regulatory mechanisms, related functional roles, or common evolutionary origins, providing further support for their classification as members of a single gene family. *MeHIPP8*, *MeHIPP25*, and *MeHIPP36* exhibited broad and relatively high expression across multiple tissues, with extensive expression across diverse cell subsets in both leaves and storage roots. This pattern suggests that these genes may represent core, broadly acting members of the *MeHIPP* family and may contribute to a wider range of biological processes. In contrast, *MeHIPP11*, *MeHIPP13*, and *MeHIPP23* displayed more restricted, cell-type-enriched expression, being detected primarily in specific tuberous root subsets, which may be related to more specialized or context-dependent functions.

In various plant species, *HIPP* genes are involved in a wide range of responses to abiotic and biotic stresses [23,24,25,26,27]. Homologous genes in *Arabidopsis* have been shown to play key roles in regulating plant organ development and mediating responses to both biotic and abiotic stresses [23,28,70]. In cassava, RNA-seq data from leaves under drought conditions and Xpm infection revealed that most *MeHIPP* genes are differentially expressed, suggesting their broad involvement in both drought and bacterial stress responses. In addition to their well-established role in heavy metal stress response, *HIPP* genes can also be targeted by pathogen effector proteins, acting as susceptibility genes in response to pathogen attack [71,72]. Under biotic stress, distinct expression patterns are observed among different HIPP gene members [71]. For instance, following infection with Xpm, *MeHIPP7-2*, *MeHIPP26*, *MeHIPP19*, *MeHIPP3-1*, *MeHIPP33*, *MeHIPP17*, and *MeHIPP35* genes were downregulated after 7 days of pathogen infection. In contrast, their expression showed an upregulation trend during infection with non-pathogenic bacteria. *MeHIPP41* displayed an initial downregulation followed by upregulation during pathogenic infection. Non-pathogenic bacterial infection resulted in its sustained downregulation.

*HIPP* genes are known to interact with drought-regulating factors, such as cyclic E3 ubiquitin ligase HOS1 and zinc finger homeodomain transcription factor ATHB29, to modulate plant responses to drought stress [28,31,73]. In cassava, genes such as *MeHIPP19*, *MeHIPP38*, *MeHIPP26*, *MeHIPP35*, *MeHIPP21*, *MeHIPP41*, and *MeHIPP8* were significantly upregulated under drought stress, with *MeHIPP21* showing the most pronounced induction. These patterns suggest that *MeHIPP21* may act as a key regulator of the drought-stress response in cassava. In contrast, *MeHIPP4-1*, *MeHIPP30*, and *MeHIPP6-2* were significantly suppressed. These findings underscore the complex and diverse expression patterns of *MeHIPP* genes in response to drought and biotic stresses in cassava. Numerous studies have demonstrated that *HIPP* genes are widely responsive to cadmium (Cd) stress, interacting specifically with Cd to play a critical role in its detoxification [21,27,61]. Our qPCR analysis revealed differential expression patterns of the six selected *MeHIPP* genes under Cd stress. Specifically, *MeHIPP8* and *MeHIPP23* were significantly upregulated, while *MeHIPP11*, *MeHIPP13*, and *MeHIPP36* were markedly downregulated. These results suggest that members of the *HIPP* gene family exhibit diverse expression patterns in response to Cd stress, likely owing to specific structural features. Overall, the expression of *MeHIPP* genes is inducible by various abiotic and biotic stresses, highlighting their potential involvement in multiple stress response functions.

## 5. Conclusions

This study presents the first pan-genome-scale analysis of the *HIPP* gene family in cassava, revealing substantial cultivar-to-cultivar variations in gene content and composition. We identified 59 *MeHIPP* pan-genes, comprising 1209 gene copies across 31 accessions. Integrative analyses of phylogeny, structural variation, selective pressure, and transcriptomic profiles elucidated the complex evolutionary history of *MeHIPPs* and their functional diversification. Bulk RNA-seq and single-cell transcriptome data demonstrated pronounced heterogeneity in *MeHIPP* expression across tissues and cell subsets, supporting potential distinct regulatory programs and divergent biological roles among family members. Moreover, transcriptomic analyses under multiple stress conditions showed that *MeHIPPs* exhibit stress-responsive expression in response to drought, Xpm, and Cd stress, underscoring their potential involvement in the adaptation of cassava to stress. Collectively, these findings expand our understanding of the cassava *HIPP* gene family.

## Data Availability

The data presented in this study are available in the article and the Supplementary Materials.

## Supplementary Materials

The following supporting information can be downloaded at https://www.mdpi.com/article/10.3390/genes17020136/s1. Figure S1: Pan-genome distribution of cassava *HIPP* genes; Figure S2: Cross-species phylogenetic tree of HIPP family; Figure S3: Phylogeny and conserved motif architecture of cassava HIPPs; Figure S4: Single-Cell Transcriptome analysis of *MeHIPPs* in cassava tuberous root and leaves. Table S1: List of the 1209 *MeHIPP* genes identified in this study; Table S2: The conserved motifs and gene structures of MeHIPP; Table S3: Distribution of cis-acting regulatory elements of *MeHIPP* genes; Table S4: Single Cell RNA-seq data of six selected genes that were used in this study; Table S5: Primers for qRT-PCR.

## Abbreviations

The following abbreviations are used in this manuscript:

Cd	cadmium
ENA	European Nucleotide Archive
GFF	general feature format version
GRAVY	grand average of hydropathy
HIPP	heavy-metal-associated isoprenylated plant protein
HMA	heavy-metal-associated
Ka/Ks	ratio of the nonsynonymous substitution rate to the synonymous substitution rate
NCBI	National Center for Biotechnology Information
OGG	orthologous gene group
qRT-PCR	quantitative real-time polymerase chain reaction
UMAP	uniform manifold approximation and projection
