# Supplementary material for: Pan-Genome Analysis and Expression Profiling of HIPP Gene Family in Cassava
Source: Genes (Basel). 2026 Jan 27;17(2):136. doi: 10.3390/genes17020136 (PMC12940333; doi:10.3390/genes17020136)
Supplement: Supplementary file 1 [file genes-17-00136-s001.zip › Figure S.pdf]

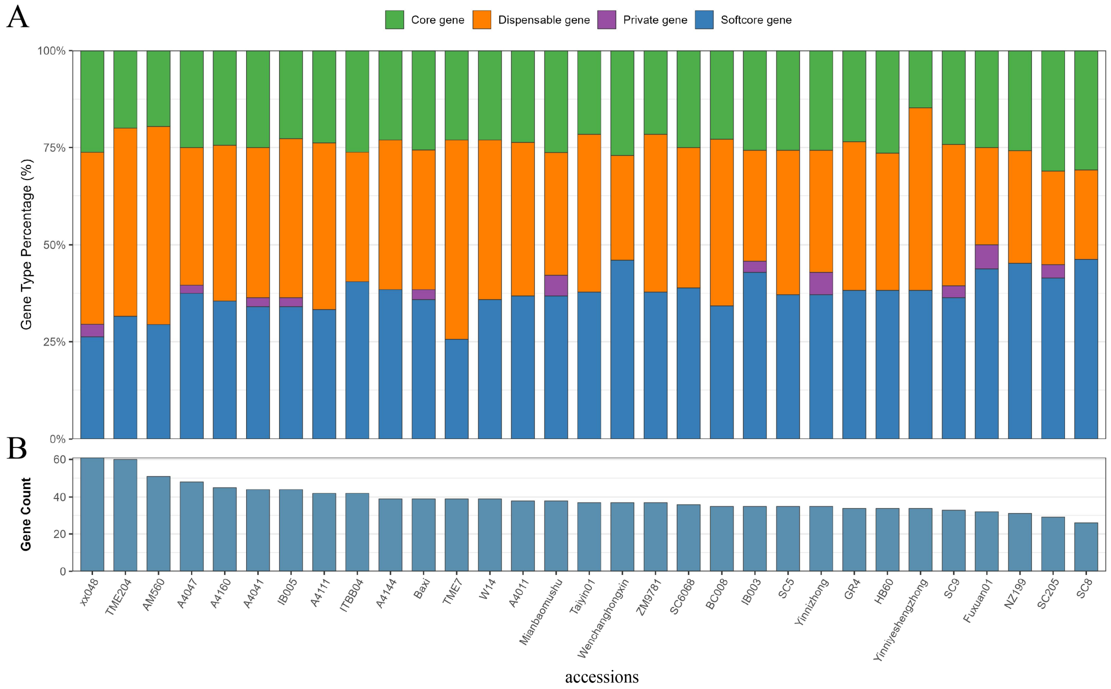

Figure S1. Pan-genome distribution of cassava *HIPP* genes. (A) Composition of *MeHIPP* gene categories (core, softcore, dispensable, and private) across 31 cassava accessions. (B) Number of *MeHIPP* genes identified per accession.

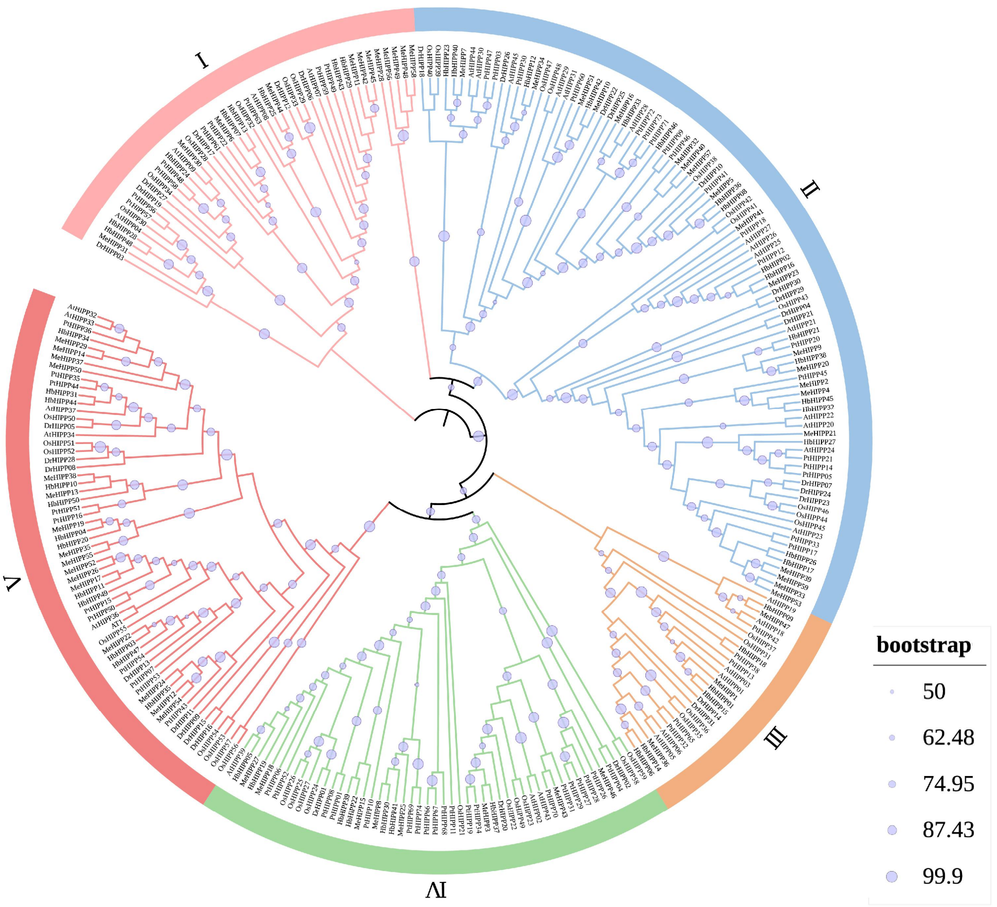

Figure S2. Cross-species phylogenetic tree of *HIPP* family, including *Manihot esculenta*, *Arabidopsis thaliana*, *Oryza sativa*, *Populus trichocarpa*, *Dioscorea rotundata*, and *Hevea brasiliensis*. The bootstrap value less than 50% were concealed. The bootstrap test was set to 1000 replicates.

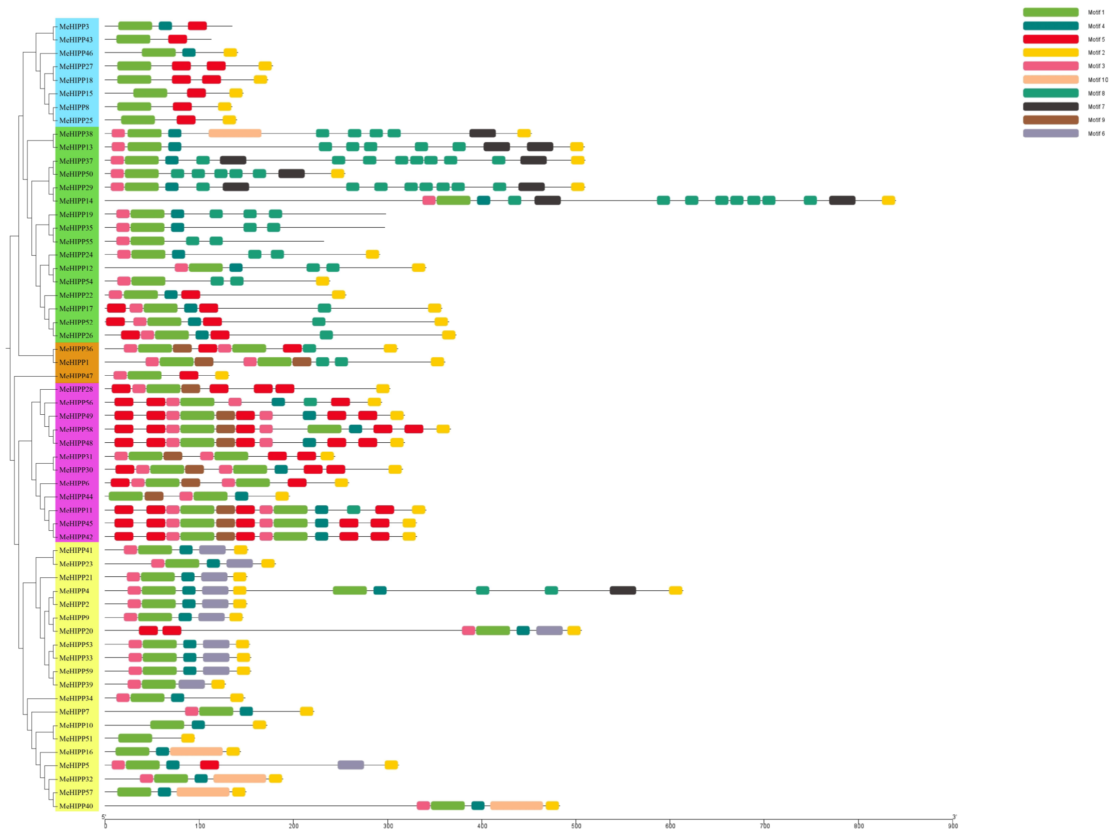

Figure S3. Phylogeny and conserved motif architecture of cassava HIPP proteins. Left panel: maximum likelihood phylogenetic tree based on HMA-domain sequences. The different groups are shown in different colors. Right panel: distribution of ten conserved motifs along each protein. motifs are represented as colored boxes and labeled Motif 1–Motif 10.

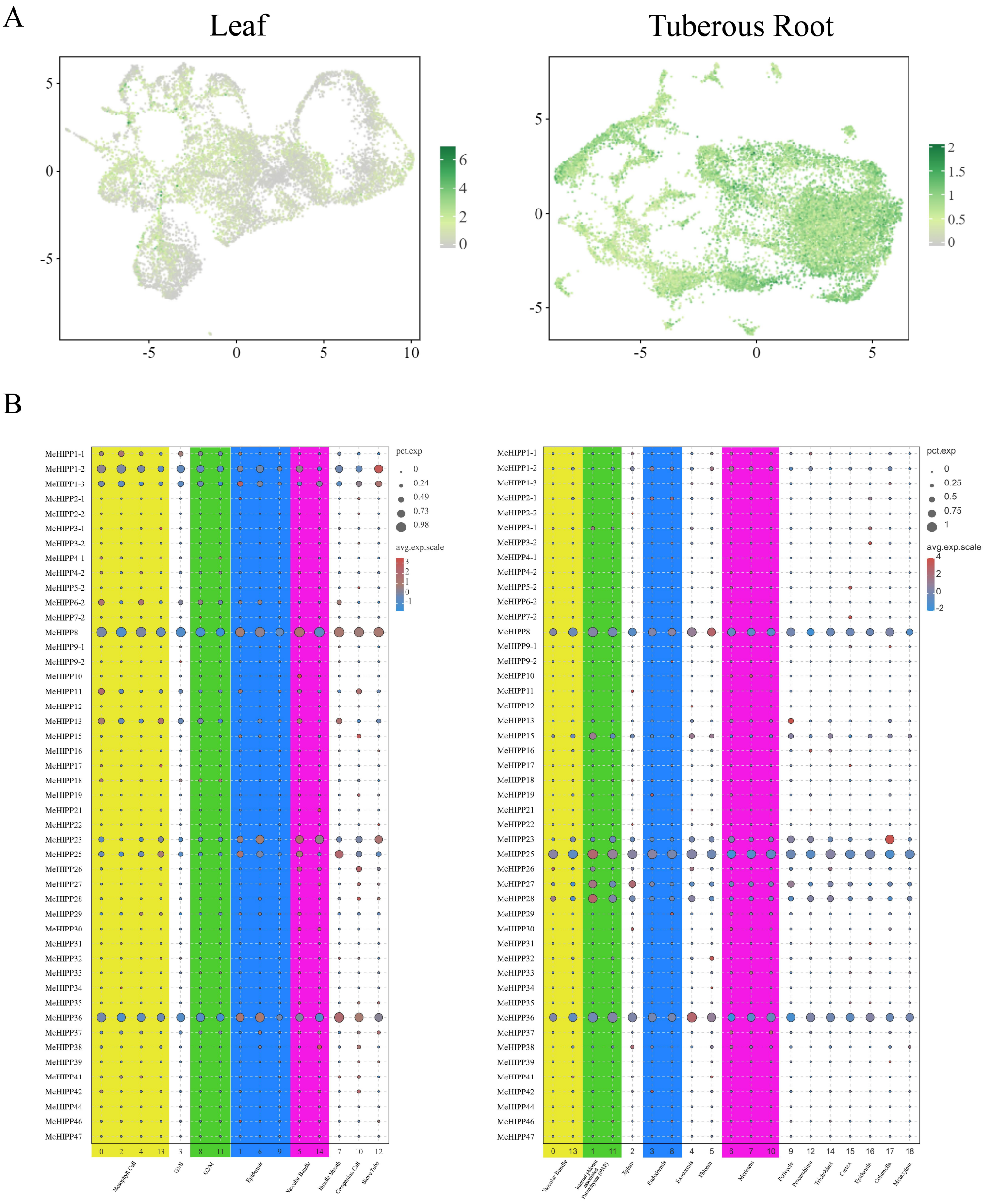

Figure S4. Single Cell Transcriptome analysis of *MeHIPP*s in cassava tuberous root and leaves. (A) UMAP of *MeHIPPs*. (B) Bubble diagram of *MeHIPPs*.
